# Meditative relaxation as an original multimodal mind-body tool: a randomized exploratory study among French hospital physicians

**DOI:** 10.3389/fmed.2026.1788066

**Published:** 2026-05-19

**Authors:** Siddhiraj Banjac, Denis Sablot, Mohamed Ali Chaouch, Coraline Lethimonnier, Alexandre Chapy, Corinne Gal, Charles Fattal, Alice Guyon, Cécile Flahault

**Affiliations:** 1 Department of Psychology, Perpignan Hospital Center, Perpignan, France; 2 Aix-Marseille University- CNRS, UMR 7077 - Center for Research in Psychology and Neuroscience, Marseille, France; 3 Department of Neurology, Perpignan Hospital Center, Perpignan, France; 4 Department of Visceral and Digestive Surgery, Monastir University Hospital, Monastir, Tunisia; 5 Department of Psychology, Montpellier Paul Valéry University, Montpellier, France; 6 Camin Team, National Institute for Research in Computer Science and Automation (Inria), Montpellier, France; 7 Laboratory of Psychopathology and Health Process UR4057, Paris Cité University, Paris, France

**Keywords:** burnout prevention, healthcare workers, meditative relaxation, mind-body intervention, psychological stress, wellbeing, non-pharmacological intervention (NPI), altered state of consciousness (ASC)

## Abstract

**Background:**

Burnout is highly prevalent among physicians and has intensified since the COVID-19 crisis. Although mindfulness-based programs can reduce stress, they often require extensive training, show variable adherence, and do not fully address emotional or existential dimensions. Meditative relaxation is a structured multimodal mind–body approach integrating attentional, sensory, physiological, and imaginative components. Its feasibility and early effects in hospital settings remain unexplored. The objective was to evaluate the short-term physiological and psychological effects of a meditative relaxation session among hospital physicians, and to compare guided meditative relaxation with an unguided active rest “do-nothing” control condition.

**Methods:**

In this randomized, single-blind exploratory trial conducted in a large public French hospital, 64 practicing physicians were assigned to either a 15-min guided meditative relaxation session (experimental group) or a 15-min unguided active rest condition (“do-nothing”) session using standardized written instructions (control group). Assessments were performed at three points: T1 (baseline), T2 (immediately post-session), and T3 (delayed by a few hours). Outcomes included physiological parameters (blood pressure, heart rate), psychological measures (SPPN, QSCPGS, SRSI3_29), and overall satisfaction (0–10 Likert scale). Qualitative feedback was collected at T3. Analyses used parametric or non-parametric tests as appropriate.

**Results:**

All participants completed the three assessment stages (*N* = 64). The sample was predominantly female (51/64, 79.7%), with a mean age of 42 ± 12 years in the experimental group and 36 ± 8 years in the control group. Overall satisfaction measured at T3 was significantly higher in the experimental group (8.11 ± 1.38) compared with the control group (6.47 ± 2.44) (Mann–Whitney-*U* = 306, *p* = 0.002, effect size = 0.40). Both groups demonstrated significant pre- to post-session reductions in systolic and diastolic blood pressure and heart rate (*p* < 0.05 and *p* < 0.01 for blood pressure), although between-group differences were not significant. Psychological assessments also improved significantly after the sessions, including reductions in stress scores and increases in global satisfaction, body satisfaction, physical relaxation, and sleepiness (*p* < 0.0001). No significant between-group differences were observed for most psychological outcomes. Internal consistency of the scales ranged from moderate to excellent, with Cronbach's α values between 0.63 and 0.89.

**Conclusion:**

A brief 15-min pause during the workday, whether guided or unguided, was associated with short-term improvements in psychophysiological indicators among hospital physicians, even if the most outcomes did not differ significantly between groups. However, guided meditative relaxation was associated with higher quantitative and qualitative satisfaction and may represent a structured and acceptable format for brief stress-management interventions. These findings remain preliminary and require confirmation in larger studies with longer follow-up.

## 1 Introduction

The WHO's 2019 ICD-11 defines burnout as a syndrome characterized by exhaustion, cynicism, and reduced professional effectiveness, representing a major public health issue (1). The prevalence among French doctors is 49%, similar to the American average, with 5% experiencing severe burnout; a systematic review of 15,183 French physicians reported high burnout rates (2, 3). These figures appear comparable across France and the US (4). Clough et al. (5) reported burnout prevalence among doctors reaching 75%, driven by increasing administrative tasks and doctor shortages. Hospital work, while meaningful, can produce stress, discomfort, and anxiety under high pressure and economic demands (6). Prolonged stress affects health across specialties, countries, and ages, leading to burnout (7). Recent studies in France highlight high burnout symptoms, stress, and suicidal ideation among hospital faculty, with physicians often underestimating their distress (8). In addition to organizational strategies and workplace adjustments, individual-focused stress prevention programs, such as mindfulness-based approaches and stress management training, are effective in reducing burnout (9, 10). Notably, mindfulness-based stress reduction (MBSR) and mindfulness-based cognitive therapy (MBCT) target mental and attentional spheres but only partly address emotional and existential aspects. Programs like Mindfulness Compassion and Insight (MBCI) or Davidson's Healthy Minds, including awareness, connection, and wisdom components, yet have limitations, such as potential neurotic awareness without solutions. Thus, there is a need for more modern, innovative techniques to prevent burnout (11). The COVID-19 crisis negatively impacted health professionals (12, 13), prompting support programs in France (14, 15). In a hospital in southern France, from 2020 to 2022, we experienced integrative mind-body support interventions for caregivers on the basis of “meditative relaxation” (16). Recent research in neurobiology (17) offers some arguments to optimize the work environment (18) to prevent burnout, suboptimal communication with patients, loss of motivation, and unprofessional behaviors. Caregivers increasingly turn to mind-body tools like yoga (19–21), relaxation (22), and meditation (23, 24), though the efficacy of meditation, mindfulness, and acceptance (MMA) depends heavily on instructor skill, method content, and participant practice (25, 26). Clough et al. (5) demonstrated that despite increasing scientific interest in examining the benefits of psychosocial/behavioral interventions on physicians' occupational stress, the quality of the research methodology did not meet expectations. However, interventions based on cognitive–behavioral principles have suggested encouraging results, and researchers have recommended in-depth evaluations, avoiding potential bias (such as instructor formation quality, content of the method, and the effectiveness of the participant practice, which is hard to evaluate), and including measures of satisfaction. Blood pressure is a significant factor in demonstrating the effectiveness of relaxation techniques (27, 28) and meditative practices appear to have longer-lasting effects on this parameter (29, 30). Multimodal mind–body approaches may offer practical tools to improve stress regulation and wellbeing among healthcare professionals. It balances relaxation and meditation, offering a straightforward, structured approach that differs from traditional relaxation or open-ended meditation by inducing a natural modification of consciousness. We hypothesized that this practice could help healthcare workers better manage professional stress, particularly physicians with limited time. We conducted a randomized, single-blind study comparing guided meditative relaxation with a simple doing-nothing approach, evaluating effects in medical doctors through quantitative and qualitative means.

## 2 Methods

### 2.1 Setting and registering

This evaluation study was conducted in a large public general hospital in southern France, employing approximately 3,500 professionals. The project was funded as part of the institution's Human Resources Department's continuing education program, within initiatives specifically supporting mental–health–related projects during the COVID-19 crisis. The conceptual framework of the study follows Phase II of the ORBIT model (31), which focuses on the preliminary refinement and evaluation of behavioral interventions through pilot randomized trials, combining quantitative, qualitative, immediate, and delayed assessments. The study design, therefore, aimed at evaluating the feasibility, acceptability, and early efficacy signals of an innovativemind–body intervention, meditative relaxation, within real-world hospital settings.

### 2.2 Participants

Participants were practicing hospital physicians representing various specialties and age groups. To be eligible, they had to be currently active clinicians in good general health, volunteer to participate during their workday, and be familiar with the use of a Device for Indirect Non-invasive Mean Arterial Pressure (DINAMAP) for measuring systolic, diastolic, and pulse blood pressure, as well as heart rate. A total sample of 64 participants (32 per group) was predetermined using GPower, based on an anticipated 18% difference in satisfaction between groups, assuming an effect size of 0.8, a standard deviation of approximately 1.8, an expected mean difference of 1.5, an alpha error of 0.05, and a statistical power of at least 0.95. All participants completed the entire study sequence (T1–T3).

### 2.3 Research design

Our *a priori* hypothesis was that although both groups would report benefits, the guided meditative relaxation session would result in significantly higher satisfaction compared with the “do-nothing” control condition as an unguided active rest condition. Delayed qualitative feedback collected at T3 was expected to provide deeper insights into individual experiences, particularly regarding elements such as perceived ease, engagement, affective response, and environmental influences (qualitative data Supplementary File 2).

Given the exploratory and pilot nature of this study, as well as a predefined analysis plan focused on theoretically grounded and pre-specified comparisons, we did not apply corrections for multiple comparisons which would have substantially reduced statistical power in our small sample; instead, we present all tests transparently, indicating the corresponding effect sizes (31, 32).

### 2.4 Randomization and blinding

The study was conducted between February and November 2022. Participants were assigned to groups using a single-blind randomization procedure, in which participants were unaware of the specific hypotheses and the meaning of group allocation. However, the investigator delivering the intervention was not blinded. Participants were randomized using sealed opaque notes containing group allocation.

- **Experimental group:** 15-min *guided* meditative relaxation (complete 3-part protocol).
- **Control group:** 15-min *unguided* session following standardized written instructions corresponding exclusively to the third component (“do-nothing”) of the full method.

The unguided active rest “do-nothing” control condition was selected to control for the potential effects of simply taking a short break during the workday, allowing exploration of the additional value of structured guidance. For the sake of accuracy, we deliberately selected a control group that was as similar as possible to the experimental group, even at the risk of creating conditions that might make it difficult to detect differences between the groups: the main differences were the specific effects of *guidance and structure* from the effects of a simple pause or mental rest. The control condition is hereinafter referred to as the “unguided active rest condition,” which takes into account the possibility that participants may have engaged in spontaneous relaxation or meditation processes.

### 2.5 Three-stage evaluation protocol

**Figure 1:**
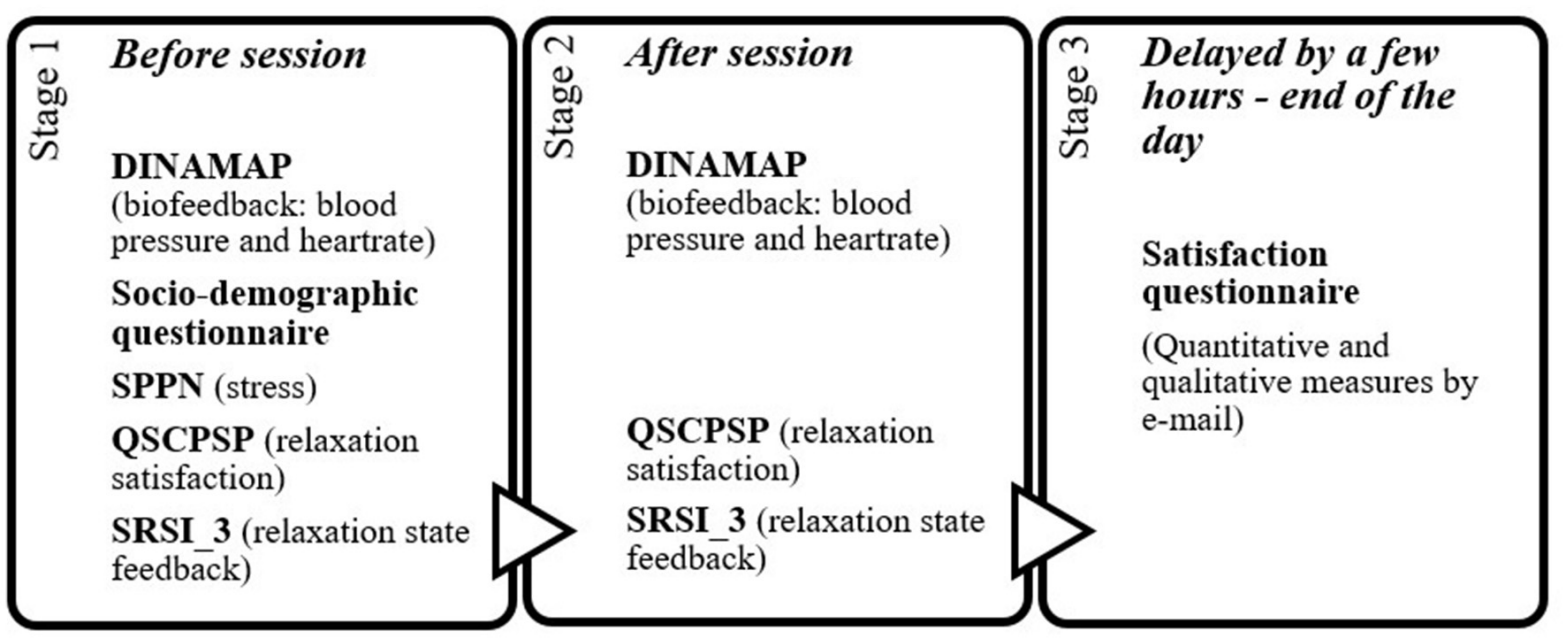
Chronological protocol for assessments.

The workflow is presented on Figure 1.

Stage 1: Pre-session assessment (T1)

Locations included:

- A psychologist's consultation office (Internal Medicine Department),
- A physician's consultation office (Rheumatology Department).

Procedures:

1. Welcome, study explanation, and informed consent.
2. Randomization by drawing lots.
3. Baseline physiological measurements using DINAMAP V100.
4. Completion of:

° sociodemographic questionnaire,
° SPPN,
° QSCPGS,
° SRSI3_29.
5. Assignment to guided or unguided sessions.

The meditative relaxation sessions differed by group allocation. Participants in the experimental group received the complete guided protocol, which included all three components of the method (Section 4). In contrast, participants in the control group completed unguided active rest session based solely on written instructions corresponding to Part 3 of the method.

Stage 2: Immediate post-session assessment (T2)

6. Completion of QSCPGS and SRSI3_29 (post-intervention).
7. Instructions about upcoming delayed satisfaction evaluation.

Stage 3: Delayed assessment (T3)

8. Conducted a few hours later, preferably at home:

- Online completion of:
- 0–10 Likert overall satisfaction score,
- qualitative open-text feedback (analyzed in Suppl. 2).

This structure enabled capturing:

- immediate psychophysiological effects (T1–T2),
- emotionally integrated reflective appraisal (T3).

### 2.6 Meditative relaxation – standardized experimental method (brief description)

(Full detailed protocol available in Supplementary File 1). “Meditative relaxation” is a three-part standardized multimodal mind–body intervention combining attentional, sensory, physiological, and imaginative components. It is intentionally structured to progressively shift attention from external sensory input to internal perceptual and interoceptive states, culminating in effortless meditative presence.

Part 1: Introductory/static elements (ETHER – EARTH)

Exercises (steps 1–6 in Suppl. 1):

- Visual focusing and defocusing
- Wide-field sensory awareness (“warrior gaze”)
- Acoustic scanning of the environment
- Spatial localization and imaginative “ascending perspective” (room → building → city → Earth → Universe)
- Return to body gravity and weight, emphasizing grounding and heaviness

This phase cultivates:

- Meta-awareness,
- Detachment from immediate work stressors,
- Grounding through somatic anchoring.

Part 2: Dynamic/interoceptive elements (AIR – FIRE – WATER)

Exercises (steps 7–13 in Suppl. 1):

- Breath observation (AIR)
- Body heat perception (FIRE)
- Heartbeat and circulatory imagery (WATER)

Each is paired with progressive muscular release “from head to toe,” repeated three times for deeper integration. Supplementary imagery, such as rivers or waterfalls, supports effortless flow and emotional regulation.

Part 3: Synthesis exercise: **unguided active rest** Exercises (steps 14–15 in Suppl. 1):

- Effortless non-doing,
- Quiet presence without judgment,
- Passive observation of internal states,
- Reintegration with gentle physical movements.

This final stage corresponds exactly to the control-group instructions but without guidance, allowing meaningful contrast between structured vs. unstructured practice.

### 2.7 Instruments

This study combined objective physiological measures, clinical self-assessment scales, and delayed qualitative evaluations. Internal consistency of the psychological instruments was evaluated using Cronbach's alpha and McDonald's omega coefficients calculated on the present sample.

#### 2.7.1 DINAMAP V100

Used to measure systolic/diastolic pressure and heart rate. Participants rested 5–10 min before measurement to ensure comparability.

#### 2.7.2 Sociodemographic questionnaire

Collected demographic information and quantified experience with relaxation or meditation using a structured scoring system (0 to 7), with additional points reflecting duration and frequency of practice.

#### 2.7.3 SPPN – positive and negative occupational stress

This questionnaire assesses levels of stimulation (SPP; 8 items) and stress (SPN; 11 items) using T-scores, which have a mean of 50 and a standard deviation of 10. Scores between 40 and 60 are considered normal, scores below 40 indicate low levels, and scores above 60 indicate high levels. The scale requires approximately 5–10 min to complete (32).

#### 2.7.4 QSCPGS – body satisfaction and global self-perception

The questionnaire contains 20 items, each scored from −5 to +5, yielding a total score ranging from −100 to +100. It evaluates two main components: body satisfaction and global self-perception (33).

#### 2.7.5 SRSI3_29 – Smith relaxation states inventory 3

The French abridged SRSI3_29 version was selected because it was the available French-language version previously translated and used at the time of study implementation. Although a more recent revision of the original instrument was subsequently published, no updated validated French version was available for this study (34, 35). Measures:

- Physical relaxation
- Sleepiness
- Positive disposition
- Spirituality/transformation
- Stressors

Responses range from 1 (“not at all”) to 6 (“maximum”).

### 2.8 Overall satisfaction

At T3, participants rated overall satisfaction with the session (0–10 Likert). They also provided qualitative explanations, which were deeply analyzed and are presented in Supplementary File 2. These rich narratives contextualize outliers (notably dissatisfied control-group participants) and clarify experiential differences between guided and unguided practice.

### 2.9 Calculations and statistics

Continuous variables were summarized as mean ± standard deviation or median [interquartile range], as appropriate. Normality assumptions were assessed using Shapiro–Wilk tests and homogeneity of variances using Levene tests. Parametric tests were used when normality (Shapiro–Wilk) and homogeneity of variance (Levene) assumptions were not violated (*p* > 0.05). Otherwise, non-parametric alternatives were applied. Paired comparisons used paired *t*-tests or Wilcoxon signed-rank tests, and between-group comparisons used independent-sample *t*-tests (with Welch correction when appropriate) or Mann–Whitney U tests as appropriate. Analyses were conducted using Excel and JAMOVI (version 2.3, 2022). Although extreme values were identified, they were retained because their exclusion did not improve data normality. Consequently, parametric tests were applied when their assumptions were met, and non-parametric tests were used when those conditions were not satisfied. Effect sizes, confidence intervals, and PRE–POST comparisons were interpreted following standard psychophysiological research conventions.

## 3 Results

**Figure 2:**
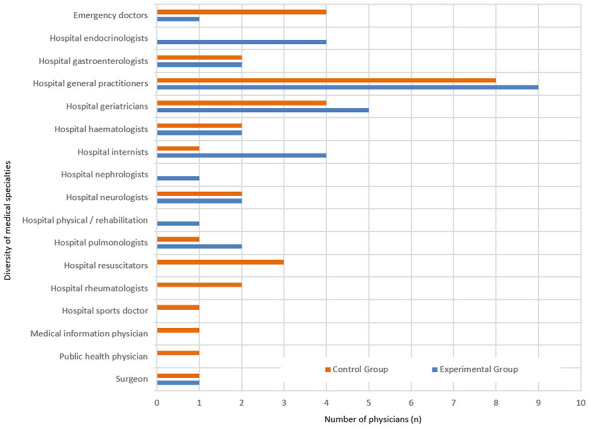
Number and diversity of medical specialties for participants of the control group (orange bars) and the experimental group (blue bars).

Sixty-four highly qualified physicians working in a French general hospital were included in the study and completed all three assessment time points (T1, T2, and T3).Thirty-two subjects in the experimental group and 32 in the control group participated in all three assessment times (T1 before the session: *stage 1*, T2 after the session: *stage 2*, and T3 delayed a few hours after the session: *stage 3*), as did the 32 subjects from the control group who participated in all three assessment times. Most of the participants were women (*n* = 51 of 64). Nine male participants in the experimental group and four male participants in the control group were randomly assigned (randomized single-blind procedure). The mean age of the experimental group (*n* = 32) was 42 ±12 years, and that of the control group (*n* = 32) was 36 ± 8 years. There were no excluded observations. Table 1 describes the characteristics of the participants. All the participants were practicing hospital doctors with high socioeconomic status: 14 were junior hospital doctors, and 50 were senior hospital doctors from various specialties and medical departments (Figure 2).

**Table 1:** Baseline characteristics of the study participants in the experimental and control groups.

Variable	Experimental (*n* = 32)	Control (*n* = 32)	*p*-value
Age (years)	41.7 ± 12.0	36.4 ± 8.0	0.07
Female, *n* (%)	23 (72%)	28 (87%)	0.18
Meditation/relaxation experience (0–7)	2.86 ± 0.45	3.72 ± 0.44	0.12

### 3.1 Overall satisfaction

**Figure 3:**
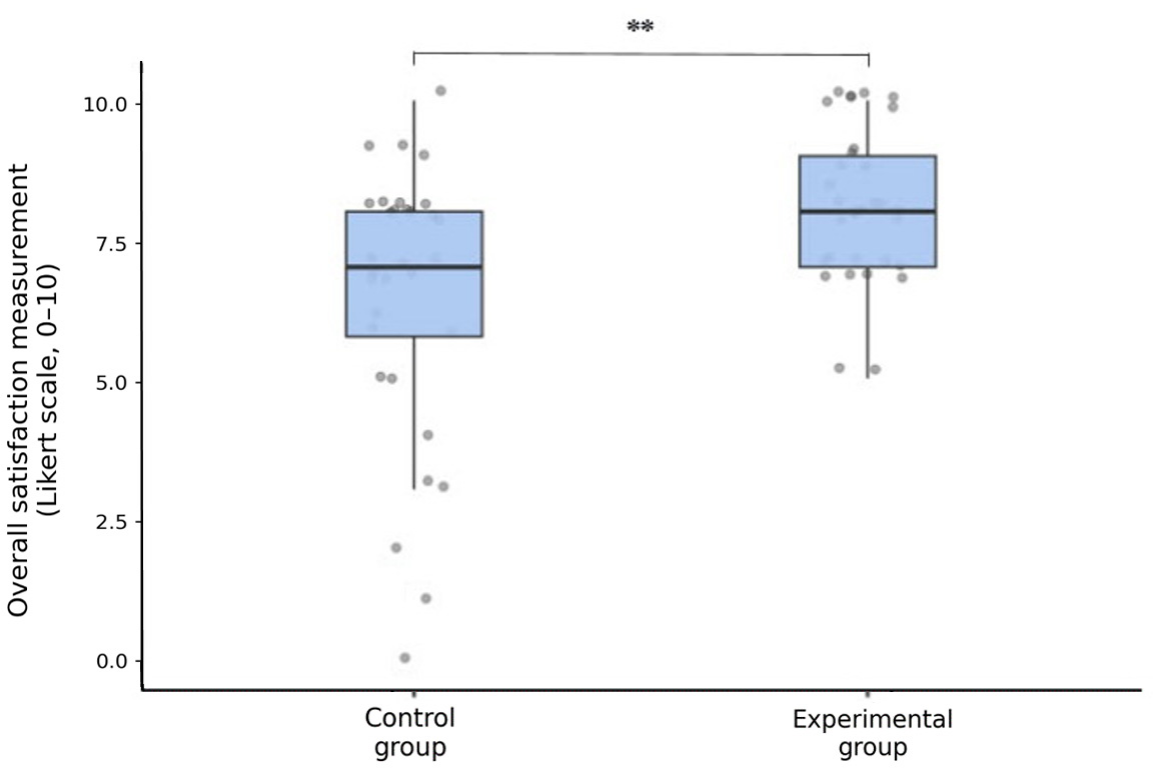
Overall satisfaction box plot measured after the intervention for the control group (*N* = 32) and the experimental group (*N* = 32). Bars show median ± IQR. ^**^p < 0.01 Outliers surrounded in red were included in the analysis and it did not change the significance.

The descriptive statistics for overall satisfaction (OS) measured by a Likert scale from 0 to 10 at stage 3 (delayed by a few hours of the session, at the end of the day) revealed a significant difference between the two groups (Figure 3). In the control group, the mean satisfaction score was 6.47 (*standard deviation* = 2.44, confidence interval 95% [5.59, 7.35]), the skewness was −1.19 (SE = 0.41), and the kurtosis was 0.82 (SE=0.81). In the experimental group, the mean satisfaction score was 8.11 [*SD* = 1.38, 95% CI (7.61, 8.61)], the skewness score was −0.29 (SE = 0.41), and the kurtosis score was −0.14 (SE = 0.81). Overall mean satisfaction was significantly greater in the experimental group than in the control group [*U* = 306, *p* = 0.002, Md = −1.00, 95% CI (−2.00, 0.00), effect size = 0.40, *N* = 64], even when the statistical tests were adapted to exclude extreme values (*U* = 306, *p* = 0.010).

### 3.2 Qualitative considerations of satisfaction ratings

To understand the three extreme values of overall satisfaction, close to 0 was obtained in the control group (*meditative relaxation* without any guidance) (Figure 3), we analyzed their qualitative feedback. Qualitative data shed light on this subject. Although the participants in the experimental group generally appreciated the relaxing effect of the practice, three participants in the control group were apprehensive about spending time “doing nothing” without guidance, as part of the unguided active rest condition (“leisurephobia” or fear to face many internal questions), which greatly diminished their satisfaction (Suppl. 2). Interestingly, these participants did not practice any relaxation, meditation, or hypnosis, whereas participants in the control group who appreciated the experience had a higher level of practice experience (Table 1).

### 3.3 Physiological parameters

**Figure 4:**
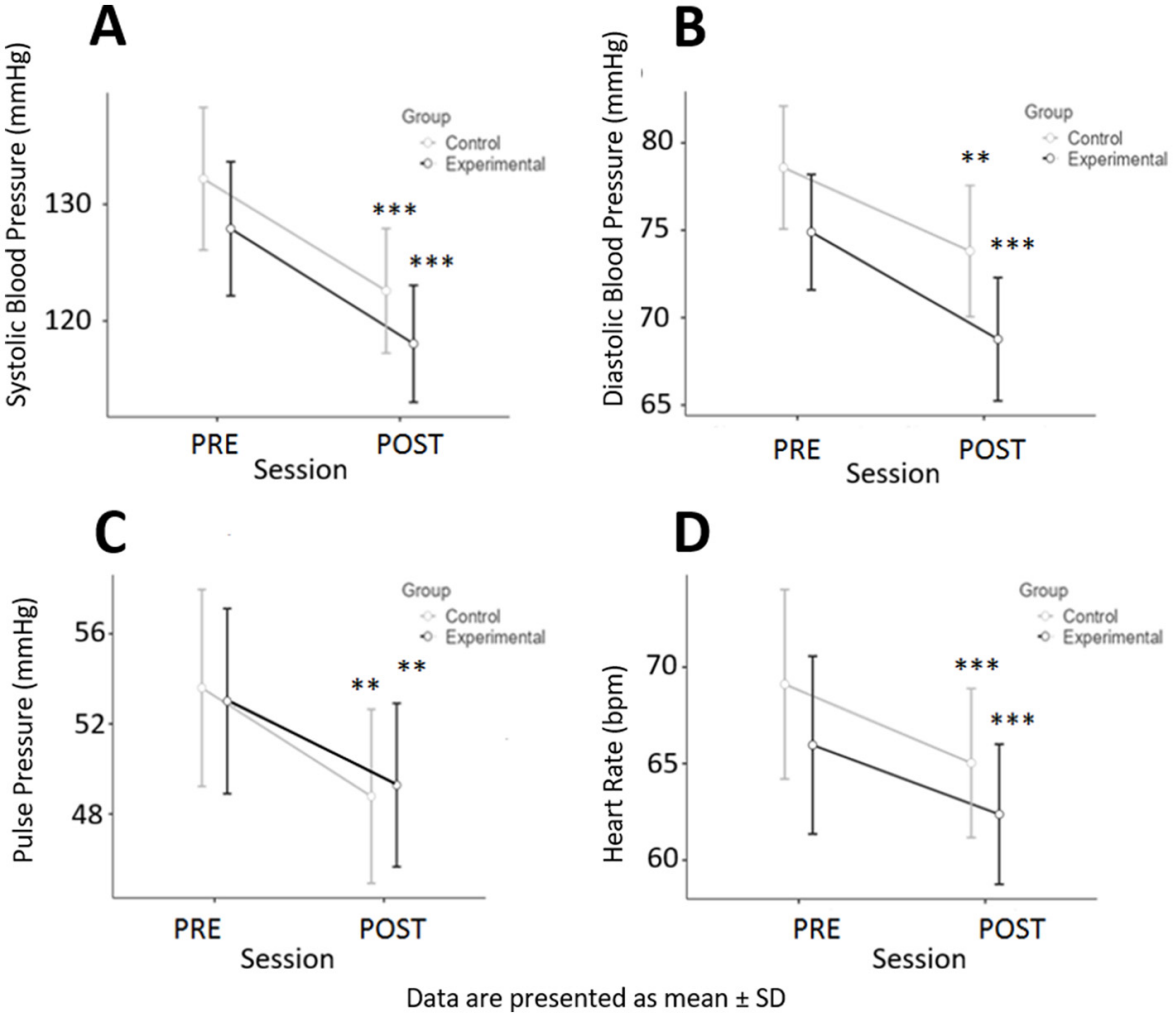
Mean comparison (with 95% CI) of physiological evaluations PRE-POST session: **(A)** Systolic blood pressure; **(B)** Diastolic blood pressure; **(C)** Pulse pressure; **(D)** Heart rate. ^**^p < 0.01, ^***^p < 0.001.

Interestingly, the participating physicians who underwent a *meditative relaxation* session achieved a significant reduction in blood pressure parameters and a significant decrease in heart rate after the session (Figure 4). However, unexpectedly, we did not observe significant differences when comparing the experimental group with the control group, as illustrated by the lack of differences in ANOVA between the groups, although ANOVA still revealed a difference between the PRE and POST values for systolic and diastolic blood pressure (*p* < 0.05 and *p* < 0.01, respectively). As there was no significant difference in baseline levels, we pooled the results of the 2 groups as presented in Table 2 (*N* = 64 participants). There was a significant effect of the 15-min sessions on all participants' physiological parameters.

**Table 2:** Changes in physiological parameters (blood pressure and heart rate) before and after the session among all participants (*N* = 64).

Parameter	Pre-session Mean ±SD	Post-session Mean ±SD	Mean difference (Post–Pre)	Test	*p*-value	Cohen's d (95% CI)
**Systolic blood pressure (mmHg)**	128.11 ± 15.60	117.86 ± 14.44	−10.25	Paired *t*-test	< 0.001	1.19 (0.87–1.51)
**Diastolic blood pressure (mmHg)**	74.84 ± 9.25	69.80 ± 10.37	−5.05	Paired *t*-test	< 0.001	0.70 (0.43–0.97)
**Pulse pressure (mmHg)**	53.72 ± 10.34	48.06 ± 9.10	−5.66	Paired *t*-test	< 0.001	0.60 (0.33–0.86)
**Heart rate (beats/min)**	69.44 ± 11.20	65.64 ± 10.50	−3.80	Paired *t*-test	< 0.001	0.73 (0.45–1.00)

### 3.4 Psychological subjective parameters measured with questionnaires

**Figure 5:**
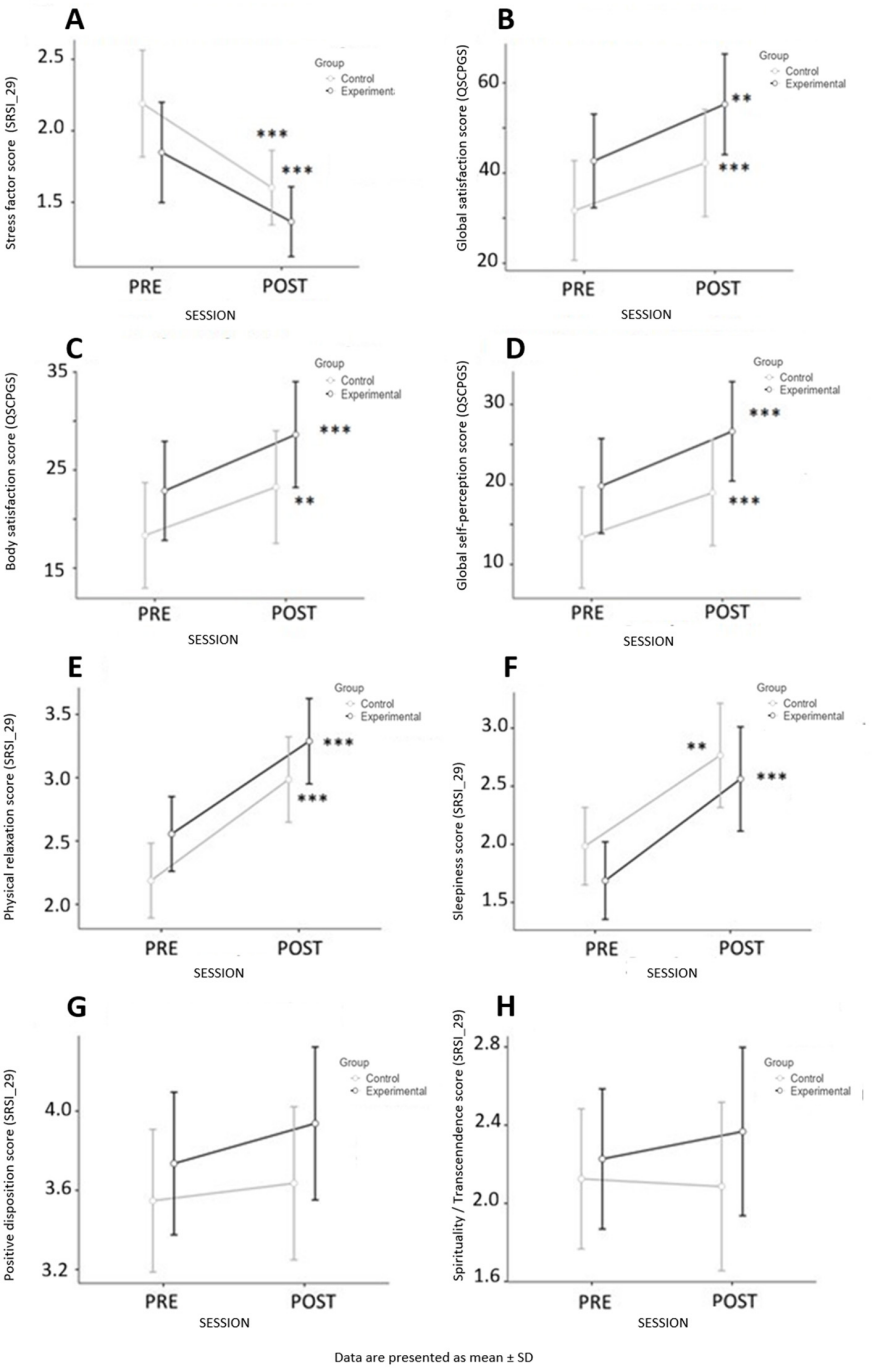
Mean comparison (with 95% CI) of psychological parameters PRE-POST session: **(A)** Stress factor (SRSI_29); **(B)** Global satisfaction (QSCPGS); **(C)** Body satisfaction (QSCPGS); **(D)** Global self-perception (QSCPGS); **(E)** Physical relaxation factor; **(F)** Sleepiness factor; **(G)** Positive disposition factor; **(H)** Spirituality/Transcendence factor. ^**^p < 0.01, ^***^p < 0.001.

Internal consistency of the psychological scales in the present sample ranged from moderate to excellent. For the SPPN scale, Cronbach's α was 0.87 (ω = 0.86), indicating excellent reliability. The SPP stimulation subscale showed lower internal consistency (α = 0.64; ω = 0.67). For the QSCPGS questionnaire, internal consistency was high (α = 0.89; ω = 0.90), with good reliability for the body satisfaction factor (α = 0.79; ω = 0.80) and the global self-perception factor (α = 0.82; ω = 0.84). The SRSI-29 total scale showed excellent reliability (α = 0.88; ω = 0.89). Subscale reliability ranged from moderate to high: physical relaxation (α = 0.87; ω = 0.87), sleepiness (α = 0.63; ω = 0.64; interpreted cautiously due to the small number of items), positive disposition (α = 0.85; ω = 0.86), spirituality/transformation (α = 0.69; ω = 0.72), and stress factor (α = 0.86; ω = 0.86). We analyzed eight different psychological parameters (Figure 5): a stress factor: the FSt (measured from SRSI_29, Figure 5A); global satisfaction, body satisfaction and global self-perception (all three measured from the QSCPGS, Figures 5B–D); a physical relaxation factor (Figure 5E); a sleepiness factor (Figure 5F); a positive disposition factor (Figure 5G); and a spirituality/transcendence factor (Figure 5H). There appeared to be no significant difference between groups for each variable before the session. Globally, as shown for the physiological parameters, we found a significant difference in PRE vs. POST in both the experimental and control groups for all the parameters tested (Figures 5A–F), except for positive disposition and spirituality/transcendence factors (Figures 5G, H). There was a significant decrease in the SRSI_29 score between the POST session and the PRE session (Figure 5A). In terms of the QSCPGS results, physical relaxation and sleepiness, the means significantly increased in the POST session compared with the PRE session for both the active and unguided active rest *meditative relaxation* groups (Student's *t* test was significant at the level of *p* < 0.0001) (Figures 5B–F). For positive disposition and spirituality factors, the effect was more subtle and was revealed only when both groups were pulled together (Figures 5G, H). Regardless of the psychological parameters tested, there was no significant difference between the experimental group and the control “doing nothing” group. As there was no significant difference in baseline levels between groups, we pooled the results of the two groups as presented in Table 3. When pooling the participants of both groups (*N* = 64), there was a significant effect of the 15 min sessions on all participant psychological subjective parameters.

**Table 3:** Changes in psychological outcomes measured using the SRSI-29 and QSCPGS questionnaires before and after the session among all participants (*N* = 64).

Outcome	Pre-session Mean ±SD	Post-session Mean ±SD	Mean difference (Post–pre)	Test	*p*-value	Cohen's d (95% CI)
**Stress factor (SRSI-29)**	2.10 ± 0.88	1.51 ± 0.44	−0.59	Paired *t*-test	< 0.001	1.09 (0.78–1.40)
**Global satisfaction (QSCPGS)**	35.92 ± 26.97	47.73 ± 28.68	+11.81	Paired *t*-test	< 0.001	0.99 (0.69–1.29)
**Body satisfaction (QSCPGS)**	20.34 ± 13.19	25.78 ± 14.03	+5.44	Paired *t*-test	< 0.001	0.80 (0.51–1.07)
**Global self-perception (QSCPGS)**	15.78 ± 15.17	21.95 ± 15.78	+6.17	Paired *t*-test	< 0.001	0.77 (0.49–1.05)
**Physical relaxation (SRSI-29)**	2.37 ± 0.85	3.14 ± 0.96	+0.77	Paired *t*-test	< 0.001	1.14 (0.82–1.45)
**Sleepiness (SRSI-29)**	1.84 ± 0.95	2.66 ± 1.26	+0.82	Paired *t*-test	< 0.001	0.65 (0.37–0.91)
**Positive disposition (SRSI-29)**	3.64 ± 1.02	3.79 ± 1.10	+0.15	Paired *t*-test	0.04	0.26 (−0.01–0.51)
**Spirituality/transcendence (SRSI-29)**	2.18 ± 1.01	2.23 ± 1.22	+0.05	Paired *t*-test	0.12	0.08 (−0.17–0.32)

SRSI-29, Smith Relaxation States Inventory; QSCPGS, Questionnaire of Body Satisfaction and Global Self-Perception.

## 4 Discussion

All 64 participants volunteered to complete the three stages of the study, each lasting 45–60 min. This point is remarkable. Overall, our study suggests that a brief structured pause during the workday may produce short-term improvements in psychophysiological wellbeing. The results of the present study related to the positive outcomes of the physiological components following the intervention provide further evidence of the potential influence of such interventions on stress reduction (27).

In a randomized controlled trial, we found that a new mind-body practice, *meditative relaxation*, which is specifically designed and used as a psychological tool in the hospital setting, improved the subjective wellbeing reported by hospital physicians and objectively assessed their physical health. Regardless of the group (“*meditative relaxation”* or unguided active rest control “*do-nothing”*), the participants achieved beneficial results after the so-called “*meditative relaxation*” session (whatever group they belonged to), in agreement with intervention reviews (33). Interestingly, there was a significantly higher degree of overall satisfaction for the treatment group than for the control group, indicating a greater advantage of this practice over an unguided active rest practice.

These results are consistent with one systematic review (34), which confirms the benefits of (meditative) relaxation in workplace-based interventions. Some statistically significant changes within-group were small in magnitude, particularly for positive disposition, and should not be interpreted as evidence of clinically meaningful improvement. In this exploratory context, effect size magnitude and consistency across outcomes are more informative than *p* values alone.

These findings should be interpreted as preliminary evidence suggesting that a brief structured mind–body intervention may be feasible and acceptable in hospital physicians. A strong argument is to propose this practice, since the strength of commitment is an important factor in the regular practice of a method that depends on subjective satisfaction and the feeling of wellbeing (35).

The unguided active rest practice group believing doing a “*meditative relaxation*” session could be carefully reflected as a kind of “placebo group.” In addition, as some participants had already experienced meditation-like practices before, it is likely that in this group some participants took advantage of the 15 min of doing nothing to rest or meditate. This is why we called this group “unguided active rest condition” and could explain why we found little differences between experimental and control groups.

Regarding objective health indicators, after a session, we observed a statistically significant drop in blood pressure and heart rate in both groups, which is a beneficial health outcome. This has already been described in the literature in connection with the practice of relaxation therapy (30) and meditative practices (29). Surprisingly, ANOVA did not reveal significant differences between the groups; however, for diastolic blood pressure, the *p* value was lower in the experimental group than in the control group (Figure 4B), suggesting that the effect might have been slightly greater in the experimental group. This similarity between the two groups regarding the effects on physiological measurements can be explained by the potential that participants may have regarded the control group as an unguided active rest or meditation practice. Indeed, such practices can also elicit relaxation responses (36) to compensate for the stress response, including decreases in heart rate (37). These physiological changes observed in both the control and the experimental groups indicate the same activation of the parasympathetic nervous system in both groups, as is often observed for both relaxation and mindfulness practices (38). Similarly, an improvement in wellbeing after the sessions was observed, especially in terms of self-reported physical (or bodily) relaxation and self-perception (39), although this improvement was not significant between groups when the comparison between the relaxation approach and the meditative approach was considered. These findings suggest that a short mind, body intervention tool of approximately 15 min can increase subjective wellbeing in a healthcare environment, providing some answers to the needs of healthcare workers (40). Increasing subjective wellbeing can lead to feeling healthier (41), and feeling healthier seems to be beneficial in situations of work-related stress, as our complementary qualitative results underline. Once again, the fact that doing nothing for 15 min achieved the same beneficial effects as active *meditative relaxation* could be because most of the participants in the control group were already experienced meditators, which could constitute bias. Indeed, for the few participants who were not meditator experts, the declared benefits were much less important, thus suggesting an advantage in guiding *meditative relaxation* over free unguided active rest sessions. However, a simple 10–15-min break seems to be beneficial for stress reduction, as a previous study has already shown (42).

Several limitations should be acknowledged.

As this pilot study involved several outcome measures without applying multiple-comparison corrections, the risk of inflated Type I error may be acknowledged. This choice aligns with our exploratory objectives and the chosen tests, but the possibility of false-positive findings cannot be entirely excluded.

Although randomization was employed, some residual imbalance remained between groups—particularly in age and sex distribution. These factors may act as potential confounders, and this limitation is inherent to small pilot samples. While additional analyses did not indicate that these variables materially altered the observed effects, their influence cannot be fully excluded.

First, the sample was predominantly female (74%), which may limit the generalizability of the findings, particularly to male physicians. In addition, the sex distribution differed somewhat between the randomized groups. Larger, adequately powered trials will be necessary to confirm these preliminary findings and control more robustly for such variables. Second, the study relied on the French abridged version of the SRSI3 (43) questionnaire translated in 2015, as it remains the only validated French version currently available. However, a more recent revision of the original instrument was published in 2021, which may limit comparability with studies using the updated version. Third, the control condition consisted of an unguided active rest (“do-nothing”) session, which may itself induce relaxation responses. Participants were instructed to rest quietly and may therefore have engaged in spontaneous relaxation or meditation practices, particularly since prior experience with meditation or relaxation was slightly higher in the control group. This may have reduced the observable differences between groups. Finally, outcomes were assessed immediately after the intervention and only a few hours later. Consequently, the present study cannot determine whether the observed effects persist over time or translate into sustained reductions in stress or burnout. Future studies including repeated sessions and longer follow-up are needed to confirm the durability and clinical relevance of these effects.

An important methodological consideration is the nature of the control condition. Participants assigned to the unguided active rest session were instructed to remain inactive for 15 min in a quiet environment. Such a condition may induce a relaxation response, which is known to produce reductions in heart rate, blood pressure, and subjective stress. Therefore, the control condition may have functioned as an active rest intervention rather than a neutral comparator, which likely contributed to the absence of significant between-group differences. Current findings suggest that a brief period of rest, whether guided or not, may be sufficient to induce measurable improvements in physiological and psychological parameters in a clinical population under significant stress mostly without significant difference between the groups; however, both quantitative and qualitative satisfaction data suggest a clear preference for guided meditative relaxation, which suggests that adherence to this type of practice may be higher than for unguided active relaxation.

Both groups ultimately contained a mind-body practice; the experimental group leaned toward relaxation, which included elements of meditation, whereas the control group was an unexpected meditation approach for participants who had already had previous personal practice. Prior experience with meditation or relaxation practices may have influenced participants' ability to engage in the unguided active rest condition, potentially contributing to the observed improvements and reducing differences between groups.” In this context, a third control group, this time without any instructions, freely organized, would be interesting to include, evaluate, and compare with the two groups presented in our study protocol. Overall, these findings should be interpreted as preliminary evidence supporting the feasibility and short-term benefits of brief rest-based interventions, rather than definitive evidence of a specific effect of guided meditative relaxation. Regarding future directions, we have found that in life, there is an often unexpected and unknown challenge that drives change and accelerates the adaptation process. In our case, it was the COVID-19 pandemic health crisis that allowed us to practice “*meditative relaxation*” on a larger scale and to offer it to French hospital teams severely affected by this crisis (44, 45). Until the present investigation, this original “*meditative relaxation*” offering had been used for years with volunteer patients, on a limited basis, in a palliative psychotherapeutic or therapeutic education setting. How can we measure the effectiveness of mind-body interventions to determine their therapeutic effects? To consider answers, we wish to project our “*meditative relaxation*” studies onto a broader research protocol in terms of participants, including group sessions, with more in-depth qualitative evaluations. Finally, to validate our evaluation, it would be relevant to include longitudinal measurements and medium- or long-term reassessments to obtain stable and reliable results and to be able to claim therapeutic efficacy.

## 5 Conclusions

This exploratory randomized study suggests that a brief 15-min pause during the workday, whether guided or unguided, may produce short-term improvements in psychophysiological wellbeing among hospital physicians. The lack of significant between-group differences suggests that the benefits observed may largely reflect the effects of a brief rest period. Further studies are needed to clarify the specific contribution of guided meditative relaxation and the putative interests for participants that have not experienced such practice before. Guided meditative relaxation was associated with higher satisfaction and may represent a structured and acceptable format for brief stress-management interventions in healthcare settings. These findings should be interpreted as preliminary and require confirmation in larger studies with longer follow-up and more clearly differentiated control conditions.

## Data Availability

The original contributions presented in the study are included in the article/Supplementary material, further inquiries can be directed to the corresponding authors.
