# Supplementary material for: Meditative relaxation as an original multimodal mind-body tool: a randomized exploratory study among French hospital physicians
Source: Front Med (Lausanne). 2026 May 19;13:1788066. doi: 10.3389/fmed.2026.1788066 (PMC13226554; doi:10.3389/fmed.2026.1788066)
Supplement: Supplementary file 1 [file Supplementary_File_1.pdf]

Supplementary document 1

“*Meditative relaxation*” – Standardized experimental method – Presentation of the detailed description

Presentation in three parts:

1 Introductory exercises/preliminary practices of the two static elements (ETHER-EARTH)

2 Basic exercises/practices of the three dynamic elements (AIR-FIRE-WATER)

3 Synthesis exercise/meditative practice of "DO-NOTHING"

---

1. Lie down comfortably, find your right position
2. Look at a specific point on the ceiling, fix, notice the color and shape (*mind focusing*)
3. Then, without moving your eyes, defocus and open your field of vision, adopt "the gaze of a warrior" who sees everything at the same time, a wide, relaxing and relaxed visual field. A meditative look that relaxes the mind. Take your time.
4. Now close the eyes and explore the sound environment; sounds, noises or silence, without judgment and without focusing
5. Awareness of where you are. Work of imagination and progressive distancing by starting in the heights; work of ***spatial localization*** and taking of "perspective of a hovering bird" and gradually gaining height by the awareness (imagination / visualization) of the building, the district, the city, the department, the landscape of the region (* see below), the regions, the country, the continent, the continents, the planet Earth, the sun, the stars leaving the Earth's atmosphere. Finally the ***awareness of the universe*** with stars everywhere in all directions.

   (*) During the time of the climb, about halfway, at the height of the department or the region, imagine at the same time 4-5 close people (family, friends, colleagues,...) active in their daily life (working, cooking, resting, shopping, driving, etc.) without attaching or lingering on a particular person. And continue the climb to the stars and the universe.

6. Return to the body, suddenly or smoothly with the feeling of ***gravitation***, gravity, weight of the ***body***, its heaviness, all the points of support on its support.

---

1 Introductory exercises or preliminary practices of the two static elements (1 – 6)

1.a Visual attention: Focusing and defocusing on the visual field

1.b Attention to the sound environment: observing all surrounding sounds and noises

1.c Spatial orientation and body awareness: Awareness of spatial localization (*ETHER ELEMENT*) and progressive imagination of distancing, back and forth: Earth-Universe-Earth. Finally, we focus on gravitation-gravity and the sensation of heaviness of the body (*EARTH ELEMENT*).

---

7. Make the connection between the outside and the inside through breathing (the ***air*** filling and emptying the lungs continuously). Observe your own natural breathing, without influence, without changing your rhythm.
8. Deepen the observation of one's breath without influence (the length of inspiration in relation to exhalation, the two moments of change between inhalation and exhalation)
9. Release the face (all muscles by gradually passing through them), neck, shoulders, arms, forearms, fingers, back, chest, stomach, hips, legs, knees, feet and through the feet in the ground – gradually let any tension disappear.
10. Be aware of the ***heat*** in your body. Feel heat levels in different parts of the body (hands, feet, belly, face, etc.) while trying to differentiate as much as possible (between body parts and between the feeling of heat or coldness).
11. With each exhalation, release the face, neck, shoulders, arms, forearms, fingers, back, chest, stomach, hips, legs, knees, feet and through the feet in the ground a second time – let any tension disappear – with each exhalation
12. Try to feel the heartbeat and heartbeat for a few moments. Imagine, visualize ***blood circulation***, blood vessels, veins and arteries, the coming and going of blood through the body (hands, feet,

legs, etc.). If it helps, then imagine a river or stream in the mountains flowing quietly and finding its own way, effortlessly; which has its own flow and environment. Which are part of nature and are similar to our blood circulation that circulates non-stop.

13. Again, and for the last time, with each exhalation release a little more, release a second time the face, neck, shoulders, arms, forearms, fingers, back, chest, belly, hips, legs, knees, feet and through the feet in the ground – disappear any tension – a little more with each exhalation. Finally, as if you were under a waterfall and it goes through you from head to toe before disappearing into the ground.

---

2 Basic exercises or practices of the three dynamic elements (7 – 13)

2.a Breathing (***AIR ELEMENT***): Observe your breathing.

- to release, relax, relieve, and let yourself fall from head to toe.

2.b Body heat (***FIRE ELEMENT***): Observe body heat; hot, cold, differences.

- to release, relax, relieve, and let yourself fall from head to toe.

2.c Heartbeat and circulation (***WATER ELEMENT***): Feel the heartbeat and visualize blood circulation, blood, water in the body, and water in nature; streams or rivers.

- to release, relax, relieve, and let yourself fall from head to toe.

---

14. Now, rest easy, ***doing-nothing***. By observing the state of the whole body, as it is, without comment... Let it be. To make the inner synthesis of this work, savoring the calm, the relaxation and every moment with oneself, to feel alive, quite simply, without having to do anything, just by being there, present. Simply doing nothing...

    Take your time.

15. Return quietly (breathe deeper, move hands, feet, and reopen eyes very slowly)

---

3 Synthesis exercise or meditative practice of "*DO-NOTHING*" (14 – 15)

Remain completely quiet during this time; stay in the moment and do nothing, with the awareness that everything takes care of itself and naturally.
