# Supplementary material for: Meditative relaxation as an original multimodal mind-body tool: a randomized exploratory study among French hospital physicians
Source: Front Med (Lausanne). 2026 May 19;13:1788066. doi: 10.3389/fmed.2026.1788066 (PMC13226554; doi:10.3389/fmed.2026.1788066)
Supplement: Supplementary file 2 [file Supplementary_File_2.pdf]

Supplementary document 2

Group N°: Overall-satisfaction-score/10, M = male or F = female, medical speciality

(Answers by email after the session at the end of the day, after work)

Experimental Group (EG)	Control Group (CG)
**EG 1: 8/10 (50 years, F, geriatrician of the hospital)**<br><br>*- "Meditative relaxation allows you to feel yourself in your body.*<br><br>*- allows me to mentally escape to my childhood*<br><br>*-It is a moment that is for me where I am with me..... "*<br><br>**EG 2: 9/10 (41 years, F, gastroenterologist)**<br><br>*- "Physical and mental relaxation*<br><br>*- Help to become aware of my self*<br><br>*- Enabled me to relativize the external problems and resulting discomfort?*<br><br>**EG 3: 8/10 (36 years, F, general practitioner)**<br><br>*"Moment of relaxation to cut with work and to focus on oneself"*<br><br>**EG 4: 7/10 (55 years, M, geriatrician of the hospital)**<br><br>*"So I think the session was not entirely effective because I couldn't let go of having to go back to my department to manage some issues that dominated and disturbed my thoughts.*<br><br>*However, I did feel a decrease in muscle tension except in the jaw. Perhaps if the*	**CG 1: 4/10 (32 years, F, hospital general practitioner)**<br><br>*"Yes, beneficial, I had 15 minutes of rest, without doing anything, without external solicitation and it still gave me some relaxation.*<br><br>*Thank you"*<br><br>**CG 2: 7/10 (36 years, F, emergency doctor)**<br><br>*"I enjoyed having a break/rest time but my thoughts were quite negative, directed towards stress or obligations. I would have liked to have been directed in this session. »*<br><br>**CG 3: 9/10 (41, F, hospital geriatrician)**<br><br>*“The benefit:*<br><br>*- feeling of being soothed*<br><br>*- body relaxation*<br><br>*- Peaceful pleasant moment*<br><br>*Thank you"*<br><br>**CG 4: 1/10 (54 years, F, hospital geriatrician)**<br><br>*"Beneficial because moment of pause but source of impatience while waiting for the end of the session"*<br><br>**CG 5: 9/10 (37 years, F, internist)**

session had been set at the end of the working day, the feeling would have been better? "<br><br>**EG 5: 9/10 (53 years, F, geriatrician of the hospital)**<br><br>*"It seems to me to have achieved something unexpected that I underestimated or did not believe possible. It is not strictly speaking a relaxation but rather a psychic and physical "inner recharge" that sleep sometimes provides. '*<br><br>**EG 6: 8/10 (36 years, F, internist)**<br><br>*"It was indeed very relaxing; however, the setting being within the walls of the hospital, it is difficult to detach from the work especially since several tasks remain to be done."*<br><br>**EG 7: 9/10 (26 years, M, rheumatology junior doctor)**<br><br>*"Very good experience, relaxing, gives serenity, Session not too long, on the other hand, some questions are quite vague"*<br><br>**EG 8: 8/10 (26 years, F, junior general physician)**<br><br>*"It is beneficial because in the end it was the only time in my working day when I took a real break and was able to relax a little and think about something other than work. Helps release muscle tension"*<br><br>**EG 9: 10/10 (39 years, M, general practitioner)**<br><br>*"- Cut a break in working time, allowing time to recharge, to ease tensions*<br><br>*- The workplace can also be associated with a place of relaxation*	*"It allowed me to clear my head and leave the stress and problems of the day aside, with a better mindset afterwards"*<br><br>**CG 6: 8/10 (37 years, F, hospital geriatrician)**<br><br>*"I think it was beneficial to me because I never thought I could really settle down at work, take time to refocus, forget the "role" to play constantly when you are in the hospital and which is tiring. Being able to let go even in the workplace allowed me tonight to let go of the constant control I put on myself.*<br><br>*So, well after I came home and I had less back pain.*<br><br>*I do not put 10 because finally it reminds me that I feel guilty to go home when I have not finished what I had to do .... And it annoys me that I can't do it more often!*<br><br>*In any case, I want to start again every day!!*<br><br>*Thank you very much 👍 👍"*<br><br>**CG 7: 8/10 (29 years, F, junior gastroenterologist)**<br><br>*" Letting go*<br><br>*Refocusing "*<br><br>**CG 8: 0/10 (37 years, F, rheumatologist)**<br><br>*"Right now I'm not in a receptive state of mind to meditation."*<br><br>**CG 9: 8/10 (37 years, F, gastroenterologist)**<br><br>*"Meditative relaxation allows us to find ourselves with ourselves, something so important almost forgotten in today's society, to focus and live in the present*

- Working on the individual also makes it possible to have a positive impact on the collective"<br><br>**EG 10: 5/10 (45 years, F, geriatrician of the hospital)**<br><br>*"I would not change my remarks since our last discussion"*<br><br>**EG 11: 5/10 (41 years, F, gastroenterologist)**<br><br>*"I don't feel that the imagination associated with relaxation has promoted relaxation for me. Although being guided, intonation timbre of voice, and recommendations... helps. I am perhaps more receptive to relaxation than to meditation.... "*<br><br>**EG 12: 7/10 (37 years, F, general practitioner)**<br><br>*"I found the session very beneficial on a personal and collective level, that is to say that by giving access to personal relaxation, we first feel relaxed and, therefore, we are more tolerant and more available to our surroundings afterwards.*<br><br>*There is a beneficial effect in the hours that follow, which is interesting, the effect is persistent beyond the session.*<br><br>*I would have appreciated the session lasting a little longer to feel completely relaxed and serene.*<br><br>*Very good antihypertensive to prescribe without moderation! "*<br><br>**EG 13: 9/10 (31 years, F, geriatric junior doctor)**<br><br>*"I found it beneficial in the sense that it allowed me to regain my positive energy."*<br><br>**EG 14: 8/10 (53 years, M, internist)**	*moment by helping us to live better with better quality of life."*<br><br>**CG 10: 6/10 (28 years, F, general medicine junior doctor)**<br><br>*"It allows you to put your thoughts in perspective because you are not distracted by the small actions of everyday life.*<br><br>*It is a real break that we do not have in a working day of 10 hours when we are junior doctor because even between noon and two the phone rings, we sit down for a very short time and, the day goes on.*<br><br>*As for me, the main action was to release my cervical muscle tension and tension headache.*<br><br>*However, I think that a workout, yoga or guided meditation are more effective. "*<br><br>**EG 11: 7/10 (28 years, F, general medicine junior doctor)**<br><br>*« Beneficial session*<br><br>*I felt relaxed and serene after the session*<br><br>*At first I was a little doubtful: "Damn I'll have to lie down doing nothing for 15 minutes, at my workplace", finally it really did me good to sit down by cutting the phones, and just to breathe*<br><br>*A time for yourself during the day feels good*<br><br>*Difficult to chase ideas related to work, but real exercise (relax / an idea that passes / chase it / etc)*<br><br>*benefit unfortunately faded in a few minutes (back in the ward, paramedics who did not understand why I did not answer the phone, pressure to see the entries, in short) Thank you! "*

"Good effectiveness of sessions with perceived muscle relaxation. Psychological relaxation was also provided, but the resumption of work in consultation just after did not allow me to fully enjoy the session."<br><br>**EG 15: 8/10 (33 years, M, internist)**<br><br>*"Beneficial session because you feel relaxed leaving the session, so it is beneficial even though the external demands are the same. Little benefit to me because I am already very relaxed basically. "*<br><br>**EG 16: 10/10 (39 years, F, pulmonologist)**<br><br>*"This session allowed me to relax both my body and my mind, gradually escaping from here and now to then make me fully reintegrate my body, with relaxation, towards a very pleasant and too exceptional final letting go. A very pleasant experience, with rapid efficiency.*<br><br>*Thank you! "*<br><br>**EG 17: 7/10 (26 years, F, physics and reeducation physician)**<br><br>*"The session tool is located in the workplace, so it is harder to relax. The principle of "escaping" from one's body is relatively effective, but the rhetorical language of the city, less relaxing. »*<br><br>**EG 18: 7/10 (61 years, M, nephrologist)**<br><br>*"The environment was not optimal, the timing was not necessarily the best, nor was my predisposition, but the session was of quality"*<br><br>**EG 19: 7/10 (42 years, F, endocrinologist)**<br><br>*"As I told you yesterday, I adhered more to the second part about the muscle relaxation of each part of the body. I reproduced the session in the evening in the quiet at home, which actually allowed me to relax a little more than in the hospital. I will try to be*	**CG 12: 9/10 (28 years, F, hospital general practitioner)**<br><br>*"Benefit felt on the feeling of anxiety on a daily basis at work. Allows you to refocus on yourself and start afresh and redefine priorities*<br><br>*Thank you"*<br><br>**CG 13: 7/10 (29 years, F, geriatric junior doctor)**<br><br>*" - meditation on the questionnaire completed just before*<br><br>*- relaxation and break in a working day*<br><br>*- Objective of the day refocused"*<br><br>**CG 14: 7/10 (32 years, F, hospital general practitioner)**<br><br>*"Meditative relaxation session is beneficial in my opinion. This is not a discovery for me. I practice it almost daily and I know it works for me. (promotes cardiac relaxation, rooting, appeasement ...)*<br><br>*The fundamental question in my opinion is how to integrate it into our professional lives... "*<br><br>**CG 15: 8/10 (31 years, F, hospital general practitioner)**<br><br>*"It allowed me to take the time, even if it's only 15 minutes you hardly take the time to stop. Attempt at a self-hypnosis session during which I dozed off at the end, with a few snippets of invasive images of hospital, which are difficult to cut off....*<br><br>*Thank you for your time! "*<br><br>**CG 16: 3/10 (40 years, F, neurologist)**<br><br>*"Given the proposal made, it was a break for me. Certainly welcome, during a standard day, but the break in rhythm*

regular and maybe even do it with children. "<br><br>**EG 20: 10/10 (55 years, F, endocrinologist)**<br><br>*"This session allowed me to relax, to get out of my work environment while taking advantage of my professional environment to replace it in a progressive way.*<br><br>*To be able to let go step by step and then relax strictly speaking.*<br><br>*It is a sequential method that has been very effective for me.*<br><br>*The fact of being guided by verbal accompaniment gives a very interesting imaginary dimension but above all it allows not to seek sleep but relaxation by letting go.*<br><br>*Very nice experience. Congratulations and THANK YOU. "*<br><br>**EG 21: 10/10 (33 years, F, general practitioner)**<br><br>*"The session was very beneficial to me. For a first experience in meditation, it really helped me to finish the working day very calm and relaxed, and I felt less tired too. "*<br><br>**EG 22: 10/10 (52 years, M, surgeon)**<br><br>*"Excellent relaxation but at a time when I was already serene and calm. I would have benefited more at a time of greater tension.*<br><br>*Very pleasant and effective moment."*<br><br>**EG 23: 7/10 (46 years, F, emergency physician)**<br><br>*"Having practiced hypnosis, it comes very close.*	*especially highlighted my background fatigue! It is difficult to consider that it is meditative relaxation, the instructions being so broad and the autonomy too great. In my opinion, a little more guidance would have helped! "*<br><br>**CG 17: 8/10 (33 years old, F, sports physician)**<br><br>*"Feeling of relaxation*<br><br>*Listening to outside noises - movements / noises of the hospital that are not paid attention to*<br><br>*Break in the busy day*<br><br>*Hypopressive breathing - "mindfulness meditation"*<br><br>*Beneficial break."*<br><br>**CG 18: 7/10 (27 years, F, rheumatology intern)**<br><br>*"The relaxation session was beneficial because it allowed you to give free rein to your thoughts, to take a break from a busy day.*<br><br>*It allows you to take 15 minutes for yourself. Maybe we lacked learning relaxation techniques."*<br><br>**CG 19: 7/10 (29 years, F, hospital general practitioner)**<br><br>*"It is beneficial especially at the end of the afternoon and after a long day of work.*<br><br>*On the other hand in my opinion, for a better relaxation it may be necessary to work on the environment even if, I know that it could be to much considering (the calm .. a more comfortable bed... More arrangement, and so on)*

It is a moment of pause where the caregiver takes the time to refocus on himself, which is too rare in my case.<br><br>*Thank you for your time"*<br><br>**GE24: 7/10 (52 years, F, hospital general practitioner)**<br><br>*"I really enjoyed disconnecting from reality and making this great and beautiful journey over the earth and into an unusual place and time: my workplace.*<br><br>*I would have liked more time to enjoy a more sustained, deeper, more lasting relaxation. But the preview was great!*<br><br>*And I noticed that more willingly I checked the boxes of "well-being" on the questionnaire after the relaxation session... intuitively...*<br><br>*Good job for the analysis! "*<br><br>**EG 25: 8/10 (27 years, F, general medicine junior doctor)**<br><br>*"- the session takes a very pleasant break from the working day - I felt rested after the session*<br><br>*- allows you to refocus on yourself"*<br><br>**EG 26: 10/10 (52 years, F, internist)**<br><br>*"When you are constantly drawn into the daily life between work and family life and you run behind time all the time, you do not necessarily think about taking a break to recharge your batteries...*<br><br>*This meditative session is undoubtedly beneficial because it allows you to escape for a few minutes elsewhere with carefree, just focus on the present moment, become aware of what surrounds us whether it is your body, your breath, your emotions, your*	*Otherwise congratulations for your effort and good luck"*<br><br>**CG 20: 6/10 (42 years, F, hematologist)**<br><br>*"-distance yourself from current events at work (care of patients)*<br><br>*- beneficial rest time*<br><br>*- probably to integrate into everyday life during work*<br><br>*- lack of practice may be with at the beginning of the relaxation work a form of panic at the idea of doing nothing"*<br><br>**CG 21: 2/10 (54 years, M, emergency doctor)**<br><br>*"Moment of relaxation imposed by the situation hence answer 2 (voluntary commitment to participate in the study). So beneficial moment in a working day but no real specificity felt in the approach except a possible self-conditioning aroused by the questionnaires. "*<br><br>**CG 22: 8/10 (26 years, F, general medicine junior doctor)**<br><br>*"- escape from everyday life*<br><br>*- reconnect with body and mind*<br><br>*- forget the hassles of everyday life*<br><br>*- ... "*<br><br>**CG 23: 10/10 (47 years, M, medical information department physician)**<br><br>*"This is the first time I have asked myself the question before and after meditation how I feel, and to see factually the beneficial impact of this practice. This confirmed my interest but it must not be a way to hide the forest... Just a solution to better live the*

sensations, or contemplate the universe and feel the lightness of gravity ...<br><br>*This exercise allows you to capture pleasant sensations and pleasant energy! "*<br><br>**EG 27: 10/10 (27 years, M, general medicine junior doctor)**<br><br>*"Big benefit in terms of energy for the rest of the day and pleasant experience of letting go"*<br><br>**EG 28: 8/10 (45 years, F, hematologist)**<br><br>*"Personally I know in theory the benefits of relaxation and meditation but I do not practice them because of my pace of life and work which does not leave me too much time to breathe. When I have free time, I choose physical activity or intellectual activities such as reading, cinema and theatre. The fact of proving a real well-being (lightness, letting go, detachment) after only 15 minutes of session gave me the desire to try again and opened the door to this practice I thought was more laborious and time-consuming. I now know it's do-able! "*<br><br>**EG 29: 9/10 (65 years, M, pulmonologist)**<br><br>*"- appeasement*<br><br>*- disappearance of oppression*<br><br>*- lightness*<br><br>*- well-being in general"*<br><br>**EG 30: 7/10 (55 years, F, hematologist)**<br><br>*"Session appreciated for several reasons: a moment of great calm and rest at the end of the day, the technique that I did not know allowing effectively in a short time to "cut" with the rhythm of everyday life, the possibility of being able to try to reproduce*	*difficult moments, not to be able to live more.*<br><br>*Thank you again for the proposal to participate."*<br><br>**CG 24: 5/10 (40 years, F, hematologist)**<br><br>*"I didn't feel like I had a real meditative relaxation session in the sense that it was brand new to me. I had no previous experience. I feel like I had trouble relaxing because I continued to be in the hospital (maybe with the sounds of the parasitic corridors). I find that 15 min is a bit short to "unhook" completely especially without outside help (music, atmosphere, voice).*<br><br>*Afterwards, these 15 minutes allowed me to release the pressure a little and I had the impression when starting to be less hunched and less tense.*<br><br>*I think that repeating "these sessions" regularly can only bring positive to the body and mind!!"*<br><br>**CG 25: 5/10 (43 years, F, public health physician)**<br><br>*"There is very little break time in a day so just stopping yourself has already done good.*<br><br>*On the other hand, I think that a more guided technique could help to achieve a more intense state of relaxation"*<br><br>**CG 26: 8/10 (35 years, F, neurologist)**<br><br>*"It has been beneficial because it allows you to refocus on yourself, reduce anxiety related to hyper-solicitations at work."*<br><br>**CG 27: 8/10 (46, M, anesthesiologist resuscitator)**<br><br>*"This relaxation session was beneficial because it created a peaceful and calm moment, just for me, without the constant*

this moment, even within a very busy day because requires only a short time."<br><br>**EG 31: 8/10 (25 years, F, neurology intern)**<br><br>*"I allow myself to send you this email to tell you that it was a concretely pleasant session, allowing a very important body relaxation, and a release of stressful energies"*<br><br>**EG 32: 7/10 (26 years, F, general medicine junior doctor)**<br><br>*“I found it difficult at the beginning of the session to focus on the lyrics without having thoughts that go to something else.*<br><br>*As the session progressed, I was able to relax and relax more. But all the same with always an effort not to have invasive thoughts.”*	*tension of being called at all times for a life-threatening emergency or a series of injured people... or other. What helped was that I was on duty and I knew that after the session I would go home. I don't know if I did a meditation relaxation session or just relaxation. “*<br><br>**CG 28: 8/10 (38 years, W, anesthesiologist resuscitator)**<br><br>*"The session brought me immediate well-being, allowing me to take a break without "aggression" (solicitations, phone etc ...) during working time. It allowed me to achieve muscle well-being and disconnect during this time. The relatively short duration makes it possible to use the method pdt work or even daily.*<br><br>*I will be curious to know the results of your study. Thank you for allowing me to participate. “*<br><br>**CG 29: 8/10 (34 years, F, pulmonologist)**<br><br>*"Work is quite stressful and scary, which for me uses a lot of my energy and necessarily influences my mental state and you forget yourself.*<br><br>*Refocusing energies on oneself and doing it more often would be ideal. “*<br><br>**CG 30: 7/10 (30 years, F, neurosurgery junior doctor)**<br><br>*"No more because I was missing music and it was at a time of the day when I still had things to do and therefore not with peace of mind like in the evening at the end of the day.*<br><br>*Otherwise it is sure that it allows you to relax. And to lower his blood pressure.*<br><br>*For me, you need a relaxation aid. A voice, a music, a mental exercise to do.*

	Interesting study, I want to know the results.<br><br>*Thank you. "*<br><br>**CG 31: 3/10 (58 years, M, anesthesiologist resuscitator)**<br><br>"I didn't really meditate. My head thought a lot of things! Then, I usually do a 15-minute "power napping" in the afternoon where I fall asleep very easily and quickly. My brain had a hard time not confusing the 'meditative session' with such a super-nap."<br><br>**CG 32: 6/10 (27 years, F, emergency junior doctor)**<br><br>"Very nice to take 15 min to just put your body down.<br><br>Difficult to put his mind on the other hand. Thoughts about the personal and somewhat professional level resurface.<br><br>Nevertheless, very pleasant moment in a working day or even a day at all<br><br>Many thanks Siddhi for this experience and for this study."
